# Investigations of Major α-Dicarbonyl Content in U.S. Honey of Different Geographical Origins

**DOI:** 10.3390/molecules29071588

**Published:** 2024-04-02

**Authors:** Kate Nyarko, C. Michael Greenlief

**Affiliations:** Department of Chemistry, University of Missouri, Columbia, MO 65211, USA; knzhb@missouri.edu

**Keywords:** honey, liquid chromatography-mass spectrometry methylglyoxal, glyoxal, 3-deoxygluscosone, geographical origins, Maillard reaction

## Abstract

α-Dicarbonyls are significant degradation products resulting from the Maillard reaction during food processing. Their presence in foods can indicate the extent of heat exposure, processing treatments, and storage conditions. Moreover, they may be useful in providing insights into the potential antibacterial and antioxidant activity of U.S. honey. Despite their importance, the occurrence of α-dicarbonyls in honey produced in the United States has not been extensively studied. This study aims to assess the concentrations of α-dicarbonyls in honey samples from different regions across the United States. The identification and quantification of α-dicarbonyls were conducted using reverse-phase liquid chromatography after derivatization with o-phenylenediamine (OPD) and detected using ultraviolet (UV) and mass spectrometry methods. This study investigated the effects of pH, color, and derivatization reagent on the presence of α-dicarbonyls in honey. The quantification method was validated by estimating the linearity, precision, recovery, method limit of detection, and quantification using known standards for GO, MGO, and 3-DG, respectively. Three major OPD-derivatized α-dicarbonyls including methylglyoxal (MGO), glyoxal (GO), and 3-deoxyglucosone (3-DG), were quantified in all the honey samples. 3-Deoxyglucosone (3-DG) was identified as the predominant α-dicarbonyl in all the U.S. honey samples, with concentrations ranging from 10.80 to 50.24 mg/kg. The total α-dicarbonyl content ranged from 16.81 to 55.74 mg/kg, with the highest concentration measured for Southern California honey. Our results showed no significant correlation between the total α-dicarbonyl content and the measured pH solutions. Similarly, we found that lower amounts of the OPD reagent are optimal for efficient derivatization of MGO, GO, and 3-DG in honey. Our results also indicated that darker types of honey may contain higher α-dicarbonyl content compared with lighter ones. The method validation results yielded excellent recovery rates for 3-DG (82.5%), MGO (75.8%), and GO (67.0%). The method demonstrated high linearity with a limit of detection (LOD) and limit of quantitation (LOQ) ranging from 0.0015 to 0.002 mg/kg and 0.005 to 0.008 mg/kg, respectively. Our results provide insights into the occurrence and concentrations of α-dicarbonyl compounds in U.S. honey varieties, offering valuable information on their quality and susceptibility to thermal processing effects.

## 1. Introduction

**Figure 1:**
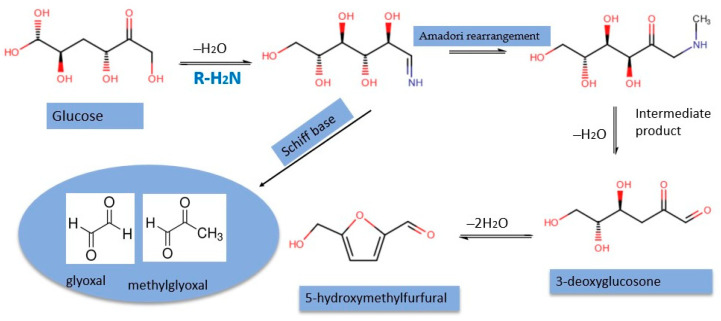
Proposed formation of common α-dicarbonyl compounds in honey.

Honey is a natural food produced by honeybees from nectar or plant secretions. The composition of honey is diverse, consisting of glucose and fructose sugars, along with water, and lower levels of minerals, vitamins, proteins, and phenolic compounds [1]. The composition of honey may differ across different honey types, creating a distinct chemical fingerprint that reflects the specific botanical and geographical origins of honey. α-Dicarbonyls are abundant in most sugar-containing foods, such as syrups, jam, candies, and bakery items, and they exhibit antioxidant activity [2]. The Maillard reaction is a common chemical reaction between amino acids and sugars that occurs during the heating process in foods. The Maillard reaction is by far the most important process that gives foods their flavor, aroma, and brown color [2]. Honey is prone to the Maillard reaction because of its high sugar content and substantial amino acid constituents. During the thermal processing of honey, the Maillard reaction occurs rapidly, leading to the formation of α-dicarbonyl compounds through a series of complex fragmentation and Amadori rearrangement reactions [3], as shown in Figure 1.

α-Dicarbonyls are reactive compounds formed during the degradation of sugars in honey through dehydration and oxidative processes [4]. Storage time, heat treatments, and processing may increase the accumulation of these compounds in honey [5]. In addition, the floral or geographical origin of the honey type, as well as factors such as pH, and temperature, can affect the formation of α-dicarbonyls in foods. The analysis of α-dicarbonyls in honey can be challenging compared to their quantification in other similar food items. This arises from honey’s complex composition, which includes sugars, proteins, amino acids, phenolics, and other constituents. Additionally, the majority of α-dicarbonyl compounds are present in honey at relatively low concentrations. Another limitation arises from the unavailability of pure α-dicarbonyl standards, which constrains the exploration of α-dicarbonyls in honey and food products. Previous research on the α-dicarbonyl composition in honey from various floral and geographical sources has revealed that methylglyoxal (MGO), glyoxal (GO), and 3-deoxyglucosone (3-DG) are some of the primary α-dicarbonyls found [6,7,8,9,10]. For instance, Marceau et al. [6] developed an HPLC-DAD method to quantify α-dicarbonyls in various types of multifloral honey and identified nine compounds, including MGO and GO derivatives. In another study, Yan et al. [11] identified nine α-dicarbonyl compounds in acacia honey. They found that 3-DG was the most abundant compound, with an average concentration of 138.09 mg/kg, and trace levels of MGO and GO ranging from 0.77 to 4.79 mg/kg and 0.56 to 3.18 mg/kg, respectively. A study by Weigel and colleagues [10] found that German multifloral honey types contain higher amounts of 3-DG compared with other α-dicarbonyls. Marshall et al. [9] also reported that Florida varietal honey types exhibited high concentrations of 3-DG, ranging from 206 to 884 µg/g with lower levels of MGO and GO identified in these types of honey.

3-DG is one of the main degradation products of glucose and acts as a precursor for the brown coloration of foods [12]. 3-DG is particularly pronounced in foods like honey due to the composition of sugars and amino acids leading to the Maillard reaction. In addition to honey, the presence of α-dicarbonyls in commonly consumed foods such as milk, beer, wine, cheese, and cookies has been extensively investigated by other researchers [7,13,14,15]. α-Dicarbonyl compounds are becoming increasingly popular as indicators for assessing honey quality and storage conditions. 5-Hydroxymethylfurfural (5-HMF), a by-product of the degradation of 3-DG, is commonly used as an indicator to assess honey quality after thermal processing and storage [8]. Moreover, despite the beneficial effects of α-dicarbonyl compounds in promoting the quality of food, high intake amounts of these compounds have been associated with chronic and age-related disease complications such as diabetes, Alzheimer’s, and cardiovascular diseases [16,17,18,19].

Numerous clinical studies have shed light on the biological implications of α-dicarbonyls and certain Maillard reaction products at in vivo levels [20,21,22,23,24]. The tissue and plasma levels of MGO, GO, and 3-DG have been shown to be significantly elevated in patients with diabetes [25]. Among all the α-dicarbonyls, 3-DG, MGO, and GO have received considerable attention because of their adverse health effects [26]. Furthermore, several attempts are being made to minimize the formation of these compounds in food products within the food industry. So far, the cytotoxic concentrations of α-dicarbonyls in honey have not been reported.

Analyzing α-dicarbonyl compounds in honey, particularly honey produced in the United States, is crucial. Methylglyoxal, for example, is well-known for its antibacterial activities and is responsible for the unique non-peroxide antibacterial activity found in Manuka honey, native to New Zealand. The investigation of this α-dicarbonyl content can help determine if similar antibacterial properties are present in honey produced in the United States. Moreover, monitoring the levels of α-dicarbonyls such as MGO, GO, and 3-DG can help ensure the quality and authenticity of honey products in the United States. High levels of these compounds might indicate improper processing or prolonged storage. While the presence of α-dicarbonyl compounds in foods is known, their occurrence in honey, specifically, in U.S. honey, has not been comprehensively investigated. In this study, we explored the α-dicarbonyl content in honey from different geographic regions in the United States and compared them to manuka honey from New Zealand to understand their role in food quality control and potential health benefits. We focused on evaluating the method validation parameters used to measure the α-dicarbonyls contents in the honey samples.

## 2. Results and Discussion

### 2.1. Identification of α-Dicarbonyls in U.S. Honeys

**Figure 2:**
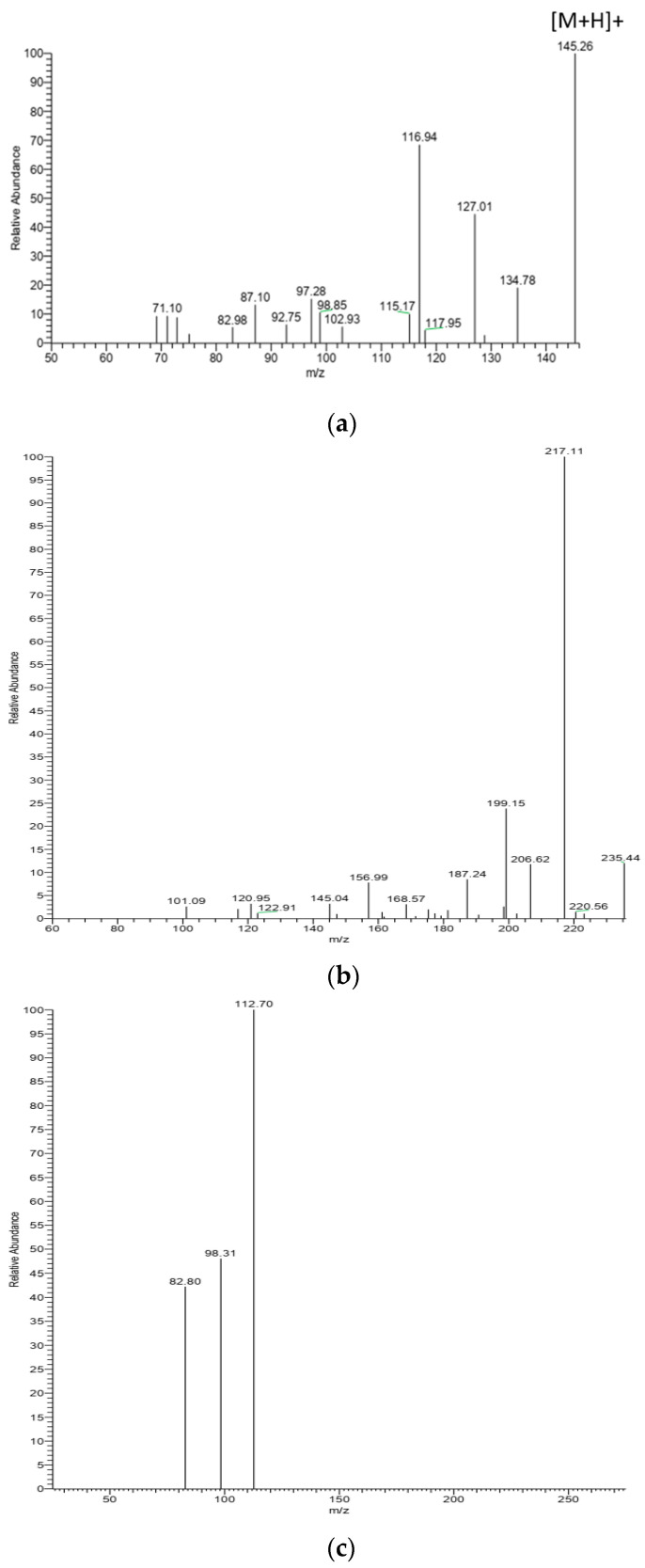
A representative HPLC-MS/MS product ion spectra of derivatized MGO, 3-DG, and GO with OPD in Washington honey [Precursor ion, (**a**) methylglyoxal, *m*/*z* = 145.26 Da, (**b**) 3-deoxyglucosone, *m*/*z* = 235.39 Da, and (**c**) glyoxal, *m*/*z* = 131.0 Da].

**Figure 3:**
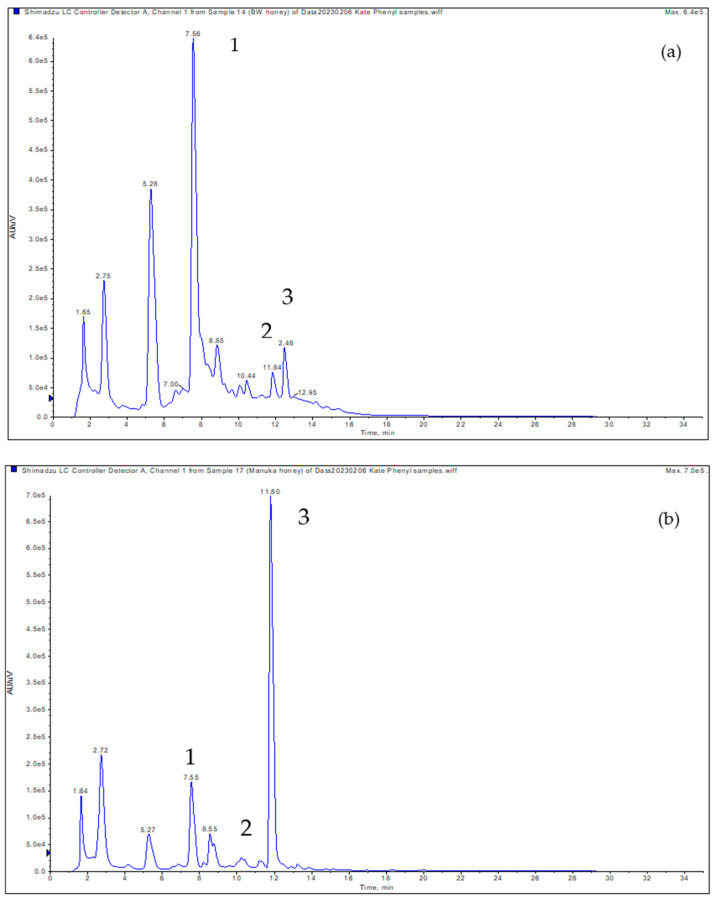
A representative HPLC-UV chromatogram of 3-DG (1), GO (2), and MGO (3) identified in (**a**) Washington honey and (**b**) Manuka honey.

α-Dicarbonyl compounds were analyzed as quinoxaline derivatives after being derivatized with o-phenylenediamine (OPD) reagent using reverse phase-HPLC in the positive electrospray ionization mode. Representative HPLC-MS/MS spectra of MGO, 3-DG, and GO derivatives are shown in Figure 2a, Figure 2b, and Figure 2c, respectively. The phenyl column used in this study improves the separation of the OPD-derivatized α-dicarbonyls in honey [13]. Methylglyoxal (MGO), glyoxal (GO), and 3-DG were tentatively identified by comparing retention times, UV spectra, and mass spectra with corresponding standards. O-phenylenediamine was employed as an effective trapping agent to selectively capture and convert MGO, 3-DG, and GO into their quinoxaline forms. In this investigation, the OPD reagent resulted in improved separation by eliminating undesired interactions with other intermediate Maillard reaction products, thereby enhancing the precise determination of α-dicarbonyls in honey. Table S1 provides information on the retention time, exact mass, molecular mass, and fragment masses of the MGO, GO, and 3-DG derivatives. The HPLC-UV chromatograms for MGO, GO, and 3-DG derivatives at a wavelength of 312 nm for Washington and Manuka honey are displayed in Figure 2a,b. Peaks 1, 2, and 3 recorded in Figure 3a,b were identified as (MGO, R_t_ = 11.80 min.), (GO, R_t_ = 10.30 min.), and (3-DG, R_t_ = 7.56 min.), respectively. Peak 4 was suspected to result from the degradation of the OPD reagent. The analysis of α-dicarbonyl derivatives in honey using HPLC, coupled with ultraviolet (UV) and diode array detection (DAD) methods, has been described in previous studies [6,8,10,27]. In our samples, we identified three principal dicarbonyls—namely, GO, MGO, and 3-DG—that have been previously detected in honey from diverse geographical locations. For instance, Weigel et al. [10] utilized a reversed-phase HPLC-UV method to analyze the α-dicarbonyl content of multifloral German honey samples. In their study, the authors identified the presence of several α-dicarbonyl compounds including GO, MGO, and 3-DG. In the analysis of Canadian honey samples using the HPLC-DAD method, GO, 3-DG, and MGO were identified among the nine α-dicarbonyl compounds by comparing their UV and collision-induced dissociation (CID) spectra with α-dicarbonyl standards [6]. Marshall and his colleagues [9] also demonstrated that the α-dicarbonyl profiles of honey from Florida contained 3-DG, MGO, and GO, as determined through HPLC-DAD-MS.

Similarly, Arena et al. [8] investigated the levels of α-dicarbonyls in various commercial honey samples from Italy using an HPLC-DAD method. Amongst all the honey studied, GO, 3-DG, and MGO were identified as the three primary α-dicarbonyls, with 3-DG being more abundant than GO and MGO. In naturally matured and artificially treated acacia honey samples collected from China, the presence of 3-DG, GO, and MGO was simultaneously detected following incubation with OPD. The analysis was carried out using UHPLC-DAD and UHPLC-ESI-Q-TOF conditions [11]. As previously mentioned, there is a dearth of information in the existing literature regarding the α-dicarbonyl content of honey in the United States. To the best of our knowledge, this study is the first to report the α-dicarbonyl concentrations of honey from different geographical origins in the United States using HPLC-UV and HPLC-MS/MS methods.

### 2.2. Optimization Conditions for Derivatization of α-Dicarbonyls in Honey

#### 2.2.1. Effects of pH on the α-Dicarbonyl Content of Honey

**Figure 4:**
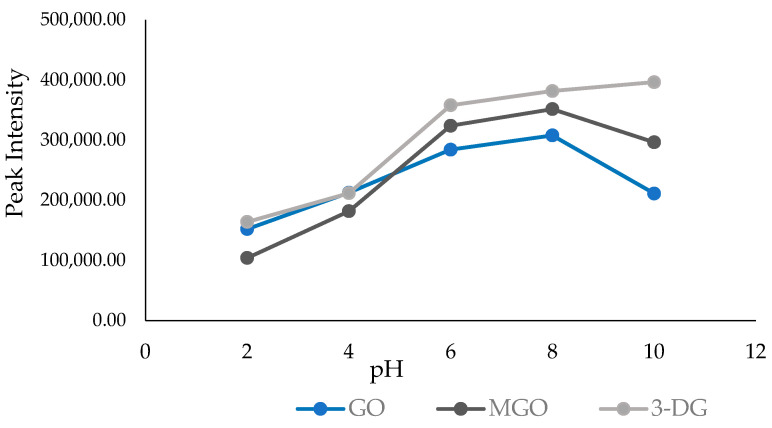
The effect of varying pH conditions on the peak intensity of GO, MGO, and 3-DG.

In the present study, we optimized the chemical derivatization reaction by assessing its efficiency under various pH conditions ranging from 2 to 10. Numerous studies have discussed the influence of pH on Maillard reaction products [28,29,30]. Overall, our findings revealed no significant correlation between the pH of the solutions and the measured total α-dicarbonyl content (*p* > 0.05, R = 0.267). The results suggest that pH might not have a direct impact on the α-dicarbonyl content of honey, although this scenario is likely under most conditions, considering the reaction conditions and the type of sample analyzed. The levels of GO, MGO, and 3-DG exhibited a corresponding decrease when solutions were prepared at much lower pH values of 2.0 and 4.0. We observed a significant influence of pH on the three target α-dicarbonyls when the reaction was conducted at a pH above 7. However, the effect of increasing pH for GO, 3-DG, and MGO appeared inconclusive. Interestingly, when we used a higher pH value of 10, we did not observe a significant increase in the GO, MGO, and 3-DG concentrations. The graph in Figure 4 visually represents the effects of pH on the levels of GO, MGO, and 3-DG in the samples.

#### 2.2.2. Effects of o-Phenylenediamine (ODP) on the α-Dicarbonyl Content of Honey

**Figure 5:**
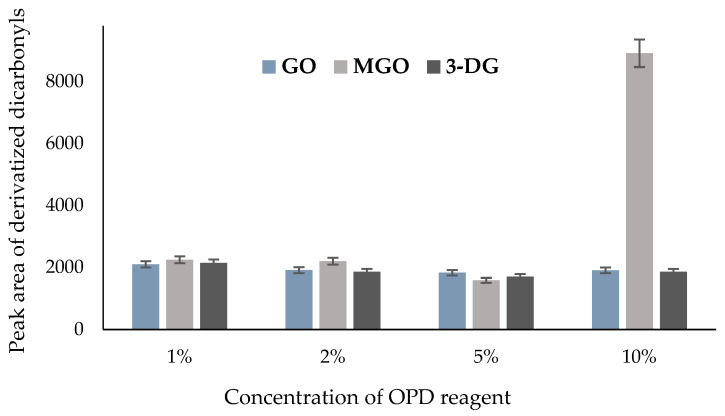
Effects of the amount of o-phenylenediamine (OPD) on the α-dicarbonyl content of honey.

The effectiveness of the derivatization reaction depends on the amount of the o-phenylenediamine (OPD) reagent. Incubations of α-dicarbonyls with OPD are typically completed within 5–24 h, depending on the steric complexity of the carbon chain structure of the α-dicarbonyl compound. Another reason may be the hemiacetal opening that occurs before the condensation reaction between the amino acid and carbonyl group during the Maillard reaction [1]. This process results in a slow rate-determining step, which decreases the reactivity time of α-dicarbonyls with the trapping reagent. This phenomenon explains why short-chain α-dicarbonyl compounds such as MGO and GO are completely derivatized in a few hours, compared with 3-DG, which requires longer hours because of its long-chain carbon backbone. In this study, we examined the effect of the trapping reagent on the detection of MGO, GO, and 3-DG in the samples. Changes in the concentrations of reagents, prepared at 0.01 g/mL (1% *w*/*v*), 0.02 g/mL (2% *w*/*v*), 0.05 g/mL (5% *w*/*v*), and 0.1 g/mL (10% *w*/*v*) concentrations, did not affect the levels of GO, MGO, and 3-DG, as illustrated in Figure 5. While the peak areas of GO and 3-DG remained unaffected, the measured peak area of MGO increased when the OPD concentration was increased to 0.1 g/mL (10% *w*/*v*). The reason behind the observed increase in MGO following incubation with 0.1 g/mL OPD reagent is not yet clear. One possible explanation is that MGO may have degraded rapidly, rendering it inaccessible for reaction with the trapping reagent, thereby requiring higher amounts of OPD for detection. Our findings here unequivocally show that an excessive OPD amount corresponds to a decrease in measured peak intensities for MGO, GO, and 3-DG. Based on these experimental results, we recommend that a lower OPD concentration between 0.01 and 0.02 g/mL (1–2% *w*/*v*) may be optimal for the determination of GO, MGO, and 3-DG in honey. Taken together, for OPD incubations, the following conditions were found to be suitable for the complete reaction of MGO, 3-DG, and GO derivatives in honey: an OPD concentration of 0.01–0.02 g/mL (1–2%) within a pH range of 7–9.

### 2.3. Method Validation for α-Dicarbonyl Determination in Honey

The quantification method was evaluated for the detection of 3-DG, GO, and MGO in the analyzed honey samples. To achieve this, various validation parameters such as linearity, precision, recovery, LOD, and the LOQ were determined. The seven-point calibration curve showed high linearity for MGO, GO, and 3-DG derivatives. The high linearity values indicate that the method exhibits linearity within the expected calibration range. The recovery was determined at three different concentration levels using a honey matrix (comprising glucose and fructose in water) and a blank. The average recovery values for MGO, GO, and 3-DG were calculated as 75.8%, 67.0%, and 82.5%, respectively, as shown in Table 1. The interday and intraday repeatability was reported as the percentage relative standard deviation (%RSD) from six replicate measurements of the honey samples. The intraday and interday %RSD values were reported to be within the range of 1.98% to 3.93% and 2.19% to 4.98%, respectively. A summary of the LOD and LOQ values reported for MGO, GO, and 3-DG is provided in Table 1. The LOD for 3-DG, MGO, and GO was calculated as 0.0018, 0.0015, and 0.002, respectively. In terms of the LOQ, the values ranged from 0.005 to 0.008. The recovery rates for GO and MGO were low, which may be due to some losses of the compounds during the wash step. However, the precision of the method was very good. The use of a solvent with a stronger elution strength may help to improve the overall recovery rates.

### 2.4. Quantification of α-Dicarbonyl Compounds in Honey

To ensure the precise quantification of α-dicarbonyls in samples like honey, it is crucial to systematically evaluate reaction parameters, including temperature, pH, amount of derivatization reagent, and reaction time. Therefore, employing highly sensitive analytical methods becomes imperative for the reliable quantification and identification of α-dicarbonyls in honey. In this study, the quantification of GO, MGO, and 3-DG in honey was based on external calibration curves established with pure quinoxaline standards, which are derivatized forms of α-dicarbonyl compounds. We evaluated the levels of the three α-dicarbonyls in a total of nineteen honey samples from Washington, Texas, and SoCal and compared them to the standard medical grade Manuka honey from New Zealand. The average levels of GO, MGO, and 3-DG in Washington, Texas, SoCal, and Manuka honey are presented in Table 2. All the samples showed increased levels of 3-DG except Manuka honey. The honey derived from SoCal demonstrated the highest 3-DG concentration, ranging from 24.5 to 50.2 mg/kg. Substantial quantities of 3-deoxyglucosone (3-DG) were also detected in the honey from Texas and Washington, with mean concentrations of 36.5 mg/kg and 17.5 mg/kg, respectively. However, we observed a statistically significant difference (*p* < 0.05, *p* = 0.008) in the individual α-dicarbonyl contents assessed across all the analyzed samples.

In another study, Degen et al. [7] investigated the α-dicarbonyl compounds in commonly consumed food products and reported higher levels of 3-DG in honey, ranging from 271 to 1641 mg/kg, and in other bakery products such as bread (13–619 mg/kg) and cookies (8.5–385 mg/kg). The increased levels of 3-DG in these food samples may be attributed to the Maillard reaction or caramelization process that takes place during food processing. The Maillard reaction typically occurs in processed foods such as honey and baked products, leading to the formation of stable advanced glycation end products, such as pyrraline, which is a major component of 3-DG [31]. The 3-DG content in monofloral honey samples as reported by Marceau et al. [6] showed a wider range, from 143 to 1099 mg/kg. Similarly, the distribution of α-dicarbonyls in Italian monofloral and multifloral honey samples revealed 3-DG as the highest α-dicarbonyl with a reported concentration of 310.8 mg/kg [8]. 3-Deoxyglucosones are known to be important intermediates in the Maillard reaction [12]. Moreover, trapping agents commonly employed in derivatization contain amine groups that catalyze the generation of α-dicarbonyls under high thermal conditions [32]. This occurrence could lead to a higher accumulation of 3-DG in the reaction mixtures when incubated with OPD and may contribute to the higher 3-DG amounts reported in numerous cases. In contrast, we detected lower levels of 3-DG in the honey samples compared with those reported in honey from various geographic regions [6,7,8,9,10]. One significant factor that may contribute to the low formation of α-dicarbonyls is the low water activity in honey, which can inhibit the initial glycosylamine reaction during the Maillard reaction [33]. The acidic pH of honey may also suppress the Maillard reaction and subsequent α-dicarbonyl formation. Furthermore, honey’s natural antioxidant properties, including the presence of flavonoids and phenolic compounds, may mitigate oxidative processes that lead to the formation of α-dicarbonyls. We also presume that the variability in honey composition could potentially explain the reduced levels of 3-DG found in the samples. This variability stems from factors like floral source, processing techniques, climate, and geographic region. Another contributing factor might be the specific floral source of the honey, as diverse floral nectars contain varying precursor levels that influence the formation of 3-DG during the Maillard reaction.

**Figure 6:**
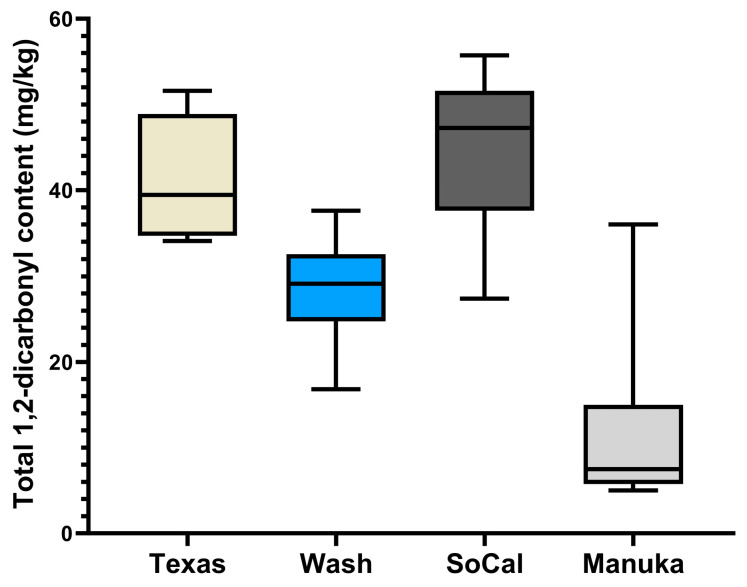
Box plot showing maximum, minimum, and median total α-dicarbonyl concentrations (MGO, GO, and 3-DG) measured in the honey of different geographic origins from Washington, Southern California (SoCal), Texas, and Manuka.

In agreement with previous findings [34,35], we recorded the highest level of MGO in Manuka honey, although this concentration was relatively lower compared with the levels reported in these studies. The average MGO content in all the samples ranged from 1.38 to 28.82 mg/kg. Furthermore, we observed lower levels of GO in Washington, Texas, and SoCal honey samples, ranging between 2.86 and 9.43 mg/kg. This range is similar to the GO levels found in honey from Florida [9], and close to the highest average concentration in multifloral honey from Italy and Hungary [8]. In contrast, GO was not detected in manuka honey, as outlined in Table 2. Figure 6 illustrates the geographical differences in the total α-dicarbonyl concentrations among the honey samples using a box and whisker plot. The plot clearly indicates that SoCal and Texas honey have a higher total α-dicarbonyl concentration compared with honey from Washington and New Zealand. The plot highlights distinct variations in individual α-dicarbonyl concentrations, providing insight into the impact of geographical origins on the composition of honey α-dicarbonyls.

### 2.5. Influence of Honey Color on the Content of α-Dicarbonyls in Honey

The color of honey plays a crucial role in complementing its origins, and it is employed to assess its quality [36]. Aging or heat treatments during processing can modify honey’s color. Moreover, prolonged heat treatments could result in increased caramelization and browning, thereby enhancing the formation of honey’s color [37]. Previous studies have suggested a correlation between the color of honey and its phenolic composition [38,39,40,41,42]. In this study, we examined the influence of color on the concentration of α-dicarbonyls in the honey samples. Our results, based on the Pfund calculations, revealed that the color of the honey samples ranged widely from light amber to dark amber tones, as shown in Table 3.

Honey produced in Texas and Washington displayed darker color tones compared with SoCal and Manuka honey, which showed significantly lighter shades. The color variation in honey is influenced by several factors including floral and geographical sources, along with processing conditions. While dark-colored honey types may be preferred for their taste or aroma, they can potentially pose a risk of extreme toxicity in humans when they contain excessive amounts of α-dicarbonyls [21,22,23,24,25,43,44]. In response to this, some studies have attempted to address the control of food browning in the food industry [45]. To date, there is currently no officially approved method to determine the color of honey; however, the Pfund color grader stands out as the most used method for this purpose. In our study, the color range of the samples fell within the acceptable range specified by the “United States Standards for Grades of Extracted Honey” [46]. We also observed a moderately positive correlation between honey color and total α-dicarbonyl concentration (R = 0.668, *p* < 0.05). While this result suggests that there is some tendency for darker honey to have higher α-dicarbonyl concentration, it does not imply a direct relationship. α-Dicarbonyl compounds formed through Maillard reactions and caramelization during honey processing and storage can occur independently of honey color and may be influenced by factors such as temperature, pH, sugar content, and the honey floral source from which the nectar is collected by bees.

### 2.6. Nutritional Consequences of MGO, GO, and 3-DG in Honey

It is worth mentioning that high concentrations of α-dicarbonyl compounds in foods are associated with the risk of chronic diseases in humans, such as diabetes, Alzheimer’s, cancer, and cardiovascular diseases [47,48,49]. Several studies have examined the presence of α-dicarbonyls in various food products and their physiological effects in humans [50,51]. Although MGO, GO, and 3-DG can be ingested through diets, the specific dose levels of each individual α-dicarbonyl that pose extreme toxicity in humans remain ambiguous. Consequently, predicting the accumulation of 3-DG, MGO, and GO in various food products and their biological impact on human cells is challenging. Furthermore, the extent to which the amount is digested and absorbed by the blood and tissues during digestion partly depends on the formation and degradation reactions that take place in intestinal cells [52,53]. The exposure of α-dicarbonyl compounds through treatment solutions and drugs has been discussed recently [54,55]. Among α-dicarbonyls in foods, 3-DG, 3,4-DGE, and 3-DGal exhibit extreme toxicity when ingested in high amounts [56,57]. In addition to the above-mentioned compounds, elevated plasma levels of MGO and GO have been linked to kidney dysfunction in uremic patients [25,58]. In the present study, we compared the concentrations of MGO, GO, and 3-DG in our samples to the average range reported in honey from different geographical origins (as presented in Table 4).

## 3. Materials and Methods

### 3.1. Chemicals and Reagents

The o-phenylenediamine reagent and quinoxaline standards were purchased from Sigma Aldrich (St. Louis, MO, USA). HPLC grade methanol was acquired from Sigma Aldrich (St. Louis, MO, USA). Sodium phosphate monobasic and sodium phosphate dibasic were purchased from Sigma Aldrich (St. Louis, MO, USA). 3-deoxyglucosone (3-DG) was purchased from Santa Cruz Biotechnology (Dallas, TX, USA). Pure methylglyoxal, glyoxal standards were procured from Sigma Aldrich (St. Louis, MO, USA).

Sep-Pak C18 cartridges (500 mg, 3 mL) for SPE were purchased from Waters (Milford, MA, USA). Syringe filters (0.45 µm, diameter 25 mm) were acquired from Sterlitech (Auburn, WA, USA). Ultrapure filtered water was obtained from a Milli-Q water purification system (Nalgene, Rochester, NY, USA).

### 3.2. Honey Samples

Twenty honey samples were analyzed in this study. A monofloral Manuka honey with a certified unique manuka factor (UMF, 20+) was purchased online from New Zealand (New Zealand Honey Co., Ltd., Wanaka, New Zealand). The UMF index is used to measure the potency of Manuka honey. A high UMF factor corresponds to an elevated level of antibacterial activity whereas a lower UMF value indicates lower antibacterial capacity. Nineteen processed multifloral honey samples in air-tight plastic containers were obtained from Washington, Texas, and Southern California in the United States. Except for the Manuka honey, the botanical origin of all the remaining honey samples was not provided. The crystallized honey samples were subjected to mild heating at 30 °C in a water bath and allowed to liquefy for 5 min. The samples were stored at room temperature in the dark until further analysis.

### 3.3. pH of Honey

To measure the pH of honey, a 50% honey solution was prepared in deionized water and subjected to mild heating at 30 °C to liquefy the crystallized honey. The pH readings of the solutions were measured using a standardized pH meter (Mettler Toledo, Thermo Fisher Scientific, Waltham, MA, USA) [63].

### 3.4. Determination of Honey Color

The color of the honey samples was classified using the Pfund procedure and formula proposed by Beretta et al. [64]. More specifically, a 50% aqueous honey solution (*w*/*v*) was prepared and subjected to mild heating at 50 °C for the complete dissolution of the sugar crystals. The measurements were carried out using a UV-Vis spectrophotometer (Agilent Technologies Inc., Santa Clara, CA, USA) at 635 nm. The absorbance readings were converted into mm Pfund values using the proposed formula in Equation (1) [65]:

mm Pfund = −38.70 + (371.39 × Abs)(1)

### 3.5. Derivatization of α-Dicarbonyls in Honey

The procedure described by Marshall et al. [9] was adopted with some modifications. α-Dicarbonyls were analyzed as their corresponding quinoxaline derivatives after incubations with o-phenylenediamine (OPD) solutions. α-Dicarbonyls are highly reactive and not easily analyzed by spectrophotometric and mass detection methods without derivatization. The OPD trapping reagent in this case prevents further reaction of MGO, GO, and 3-DG with undesirable constituents in honey. First, 1 mL of 15% (*w*/*v*) honey in sodium phosphate buffer (0.1 M, pH = 6.5) was mixed with 0.6 mL of 1% (*w*/*v*) OPD reagent in 0.1 M sodium phosphate buffer. The honey solutions were kept in the dark at room temperature for 12–24 h to ensure the completeness of the reaction. Following the derivatization step, the honey solutions were incubated and filtered through a 0.45 µm pore membrane filter. Subsequently, the filtrate obtained was taken through a solid phase extraction (SPE) process to remove excess derivatization products. SPE was performed using C18 cartridges (Sep-Pak, 500 mg, Waters, Milford, MA, USA).

### 3.6. Preparation of α-Dicarbonyls (MGO, GO, and 3-DG) in Honey

Pure quinoxaline standards for MGO and GO named as MGO-Q and GO-Q, respectively, were obtained and prepared in 80% methanol at 0.145 mg/mL and 0.06 mg/mL, respectively. The working solution was prepared by dilution with 10% methanol. The calibration ranges for MGO-Q (0.145–0.001 mg/mL), GO-Q (0.03–0.001 mg/mL), and 3-DG standard (5–0.08 mg/mL) were used for quantitative analysis. In total, 2 mL of each standard solution was derivatized using the same procedure described previously for the honey samples, and 10 µL of the standard solution was injected for HPLC-MS analysis.

### 3.7. HPLC-MS/MS Analysis of α-Dicarbonyl Derivatives

The three α-dicarbonyl derivatives were separated by a reverse-phase high-performance liquid chromatograph (Perkin Elmer, Shelton, CT, USA) coupled to an LCQ Deca XP+ ion trap mass spectrometer (Thermo Finnigan, San Jose, CA, USA). The separation was achieved on an Inert sustain phenyl column (5 µm × 2.1 mm × 150 mm, GL Sciences, Torrance, CA, USA) maintained at a temperature of 40 °C. A binary solvent system consisting of mobile phase A (water with 0.1% formic acid) and mobile phase B (methanol with 0.1% formic acid) was operated under the following gradient conditions: 3 min, 5% B, 3–8 min,40% B; 8–18 min, 100% B; 18–24 min, 20% B; and 24–30 min, 5% B for column equilibration run time. Then, 10 µL of the derivatized honey solution was injected at a flow rate of 0.3 mL/min into the LC system for a total run time of 30 min. The identification of α-dicarbonyl compounds in the honey samples was confirmed by comparing their retention times and MS/MS mass spectra with the standard solutions. Mass spectrometry conditions were performed on an ion trap mass spectrometer using an electrospray ionization source operated in the positive mode. The ESI source was set using the following conditions: capillary temperature of 320 °C and an ionization voltage of 4.5 kV. MS spectra for both standards and honey samples were acquired in the full scan mode within the *m*/*z* range of 100–500 Da. The MS/MS fragmentation studies for each analyte were assisted using a data-dependent scan with nominal collision energies set between 20 and 40 eV and He as the collision gas. LC-MS/MS data acquisition and processing was executed using Xcalibur software, version 2.0.7 (Thermo Finnigan, San Jose, CA, USA).

### 3.8. HPLC-UV Analysis of α-Dicarbonyl Derivatives

A Shimadzu HPLC 20AD pump system coupled with a UV-Vis detector SPD 20 AV (Shimadzu, Columbia, MD, USA) was used for the determination of α-dicarbonyl compounds. The separation was achieved on an Inert sustain phenyl column (5 µm × 2.1 mm × 150 mm, GL Sciences) maintained at a temperature of 40 °C. A binary solvent system consisting of mobile phase A (water with 0.1% formic acid) and mobile phase B (methanol with 0.1% formic acid) was operated under the same gradient conditions as described previously. The analytes were monitored at 312 nm, and the UV spectrum was recorded from 200 to 400 nm.

### 3.9. Quantitation of Derivatized α-Dicarbonyls in Honey

The quantification of MGO, GO, and 3-DG derivatives was based on an external calibration curve for MGO-Q, GO-Q, and 3-DG standards, respectively. The quantification of MGO, GO, and 3-DG derivatives in the honey samples was based on the peak areas of the analytes and the quotients of the areas of the analytes and respective standards. Replicate measurements were carried out on each sample. The quinoxaline concentration was expressed as mg/kg.

### 3.10. Method Validation for α-Dicarbonyl Analysis

#### 3.10.1. Linearity

The linearity was determined by linear regression analysis. A seven-point calibration was created for MGO-Q, GO-Q, and 3-DG standards and covered the concentration ranges. The calibration plot was based on the analyte response against each standard concentration for MGO-Q, GO-Q, and 3-DG. The linear fit for the curve was estimated from the linear regression analysis.

#### 3.10.2. Accuracy/Recovery

A recovery experiment was conducted to evaluate the accuracy of the method for the quantitation of MGO, GO, and 3-DG derivatives in the honey samples. A honey matrix and a blank solution were prepared and spiked with MGO, GO, and 3-DG standards at three different concentration levels, as shown in Table 5. For each spiked concentration level, the samples were independently analyzed in triplicate measurements. The mean recovery of MGO, GO, and 3-DG were expressed as follows in Equation (2).

(2) $$Recovery=\frac{\left[\;\left(concentration\;of\;analyte\;in\;honey\;matrix\right)-\left(concentration\;of\;blank\right)\right]}{Amount\;of\;analyte\;added}\times 100\%$$

#### 3.10.3. Precision

Precision was determined by running six replicate measurements of each honey sample analyzed independently on three consecutive days (*n* = 18). The precision was expressed as the percent relative standard deviation (%RSD).

#### 3.10.4. Limit of Detection (LOD) and Limit of Quantitation (LOQ)

LOD and LOQ values for MGO, GO, and 3-DG were estimated from the linear calibration curve based on the standard deviation of the response (Sy) and the slope of the calibration curve. The LOD and LOQ of the method were calculated from Equation (3):

(3) $$LOD=3.3\times \left(\frac{Sy}{Slope}\right)\;and\;LOQ=10\times (\frac{Sy}{Slope})$$

#### 3.10.5. Statistical Analysis

The average concentrations of GO, MGO, and 3-DG were expressed as (mean ± SD) values derived from replicate measurements and analyzed by GraphPad Prism 10.0. A one-way analysis of variance (ANOVA) was used to estimate the mean differences in the MGO, GO, and 3-DG content among the Washington, Texas, SoCal, and Manuka honey samples. Pearson’s correlation was employed to estimate the relationship between the reaction conditions and the MGO, GO, and 3-DG contents. Statistical significance was determined as a *p*-value < 0.05.

## 4. Conclusions

In this study, we conducted a comprehensive assessment of α-dicarbonyl concentrations in honey samples from various regions across the United States. Our findings shed light on the occurrence and levels of MGO, GO, and 3-DG as major α-dicarbonyls in these honey samples. In this study, 3-DG was found as the predominant α-dicarbonyl across all U.S. honey samples, with concentrations ranging from 10.80 to 50.24 mg/kg. The total α-dicarbonyl content varied between 16.81 and 55.74 mg/kg, with Southern California honey exhibiting the highest concentrations. Through our investigations, we elucidated several factors influencing the presence of α-dicarbonyls in honey. The pH of the honey solutions did not show a significant correlation with the total α-dicarbonyl content, indicating that other factors might play a more substantial role in their formation. Additionally, we found that lower amounts of the OPD reagent were optimal for efficient derivatization of MGO, GO, and 3-DG in honey. Comparing our results with standard medical grade Manuka honey from New Zealand, we observed varying levels of α-dicarbonyls across different U.S. honey varieties. Particularly, SoCal honey exhibited the highest 3-DG concentration among the samples studied. Geographical variations were evident in this study, with the SoCal and Texas honey samples showing higher total α-dicarbonyl concentrations compared with the Washington and Manuka honey samples. The lower levels of 3-DG found in our samples compared with other studies could be attributed to various factors such as honey composition variability. Our findings emphasize the need for further exploration into the factors influencing α-dicarbonyl formation in honey. Additionally, understanding the levels of α-dicarbonyls in honey is crucial, given their potential implications on food quality, processing effects, and human health. The variations observed in MGO, GO and 3-DG concentrations among the different honey varieties in our study demonstrate the importance of region-specific studies and quality assessment in honey production.

## Figures and Tables

**Table 1:** Method validation parameters for MGO, GO, and 3-DG.

α-Dicarbonyl	Linearity (R^2^)	LOD (mg/kg)	LOQ (mg/kg)	Intraday Precision (%RSD)	Interday Precision (%RSD)	%Recovery (Mean ± SD)
MGO	0.9989	0.0018	0.006	1.98	2.19	75.8 ± 0.21
GO	0.9999	0.0015	0.005	2.68	4.98	67.0 ± 0.21
3-DG	0.9979	0.002	0.008	3.93	4.24	82.5 ± 0.25

**Table 2:** The concentration of MGO, GO, and 3-DG in honey.

Honey	Geographical Location	3-Deoxygluconse (3-DG) mg/kg	Glyoxal (GO) mg/kg	Methylglyoxal (MGO) mg/kg	Total Dicarbonyl Content (mg/kg)
Washington A	Washington	10.80	3.44	2.57	16.81
Washington B	Washington	22.14	9.43	6.03	37.60
Washington C	Washington	18.56	8.10	4.25	30.91
Washington D	Washington	17.02	9.26	1.38	27.66
Washington E	Washington	21.04	7.88	1.69	30.62
Washington F	Washington	15.71	7.42	4.23	27.36
Texas A	Texas	30.26	4.61	N/D ^1^	34.87
Texas B	Texas	29.12	4.94	N/D	34.07
Texas C	Texas	46.99	4.59	N/D	51.58
Texas D	Texas	37.47	4.62	N/D	42.09
Texas E	Texas	43.34	4.65	N/D	48.00
Texas F	Texas	32.04	4.76	N/D	36.80
SoCal A	Southern California	24.51	2.86	N/D	27.38
SoCal B	Southern California	36.24	4.75	N/D	41.00
SoCal C	Southern California	41.37	5.56	N/D	46.94
SoCal D	Southern California	45.45	4.74	N/D	50.19
SoCal E	Southern California	42.84	4.75	N/D	47.60
SoCal F	Southern California	50.24	4.49	N/D	55.74
Manuka	New Zealand	8.16	N/D	28.82	36.98
Average		30.17 ± 12.97	5.60 ± 1.93	6.99 ± 9.76	38.12 ± 10.16
Range		10.80–50.24	2.86–9.43	1.38–28.82	16.81–55.74

^1^ N/D—not detectable.

**Table 3:** Effects of honey color on α-dicarbonyl content of honey.

Honey	Geographical Origin	pH	Color	Total Dicarbonyl Content (mg/kg)
Washington A	Washington	3.80	89.94 (Light amber) ^1^	16.81
Washington B	Washington	3.81	89.94 (Light amber)	37.60
Washington C	Washington	3.83	90.45 (Light amber)	30.91
Washington D	Washington	3.74	90.84 (Light amber)	27.66
Washington E	Washington	3.83	91.11 (Light amber)	30.62
Washington F	Washington	3.81	90.76 (Light amber)	27.36
Texas A	Texas	4.20	117.69 (Dark amber)	34.87
Texas B	Texas	4.00	117.31 (Dark amber)	34.07
Texas C	Texas	3.75	118.27 (Dark amber)	51.58
Texas D	Texas	3.73	118.42 (Dark amber)	42.09
Texas E	Texas	3.74	119.86 (Dark amber)	48.00
Texas F	Texas	4.05	120.44 (Dark amber)	36.80
SoCal A	California	3.88	99. 97 (Amber)	27.38
SoCal B	California	3.90	100.56 (Amber)	41.00
SoCal C	California	3.82	100.53 (Amber)	46.94
SoCal D	California	3.83	99.42 (Amber)	50.19
SoCal E	California	3.86	100.77 (Amber)	47.60
SoCal F	California	3.96	99.76 (Amber)	55.74
Manuka	New Zealand	3.87	64.42 (Light Amber)	36.98
Average		3.86 ± 0.11	100.92 ± 14.58	38.12 ± 10.16
Range		3.74–4.20	64.42–119.45	16.81–55.74
Regression values		R = 0.267 (*p* > 0.05)	R = 0.668 (*p* < 0.05)	

^1^ Pfund value used to determine color.

**Table 4:** Reported α-dicarbonyl content range in honey of different floral and geographical origins.

Honey	Geographical Origin	MGO (mg/kg)	GO (mg/kg)	3-DG (mg/kg)	References
Multifloral	Germany	0.4–5.4	0.2–2.7	79–1266	[10]
Multifloral	Canada/Australia/Hungary	0.8–33	0.3–1.3	143–1099	[6]
Multifloral	Italy/Hungary	0.2–2.9	0.1–10.9	75.9–808.8	[8]
Multifloral	Florida/New Zealand	3.63–483	2.19–7.35	206–884	[9]
Manuka	New Zealand	N/D ^1^–761	N/D–7.0	119–1451	[35]
Manuka/Revamil	New Zealand/Netherlands	29.3–497.1	14.4–27.3	221.6–687.3	[59]
Multifloral	Multiple locations	1.6–725	N/D	271–1641	[7]
Acacia	China	0.77–4.79	0.56–3.18	114.36–146.42	[60]
Multifloral	Spain	N/D	N/D	150–2380	[61]
Honeydew	Italy	5.7–9.9	N/D	N/D	[62]
Eucalyptus	Italy	9.9–12.7	N/D	N/D	[62]
Multifloral	USA	1.38–28.82	2.86–9.43	10.80–30.24	[62]

^1^ N/D—not detected.

**Table 5:** Spiked concentrations of MGO, GO, and 3-DG added to the honey matrix and blank for recovery analysis.

α-Dicarbonyls	Low Concentration (mg/mL)	Middle Concentration (mg/mL)	High Concentration (mg/mL)
Spiked MGO	0.001	0.01	0.1
Spiked GO	0.006	0.03	0.06
Spiked 3-DG	0.001	0.01	0.1

## Data Availability

Original data sets are available upon request to the corresponding author.

## Supplementary Materials

The following supporting information can be downloaded at: https://www.mdpi.com/article/10.3390/molecules29071588/s1, Table S1: Retention time, exact mass, molecular mass, and fragment masses of MGO, GO, and 3-DG derivatives. Table S2: Information on honey sample collection. Figure S1: PCA plot showing the geographical origin variations for U.S. honey samples and Manuka honey from New Zealand.
