# Supplementary material for: Investigations of Major α-Dicarbonyl Content in U.S. Honey of Different Geographical Origins
Source: Molecules. 2024 Apr 2;29(7):1588. doi: 10.3390/molecules29071588 (PMC11013281; doi:10.3390/molecules29071588)
Supplement: Supplementary file 1 [file molecules-29-01588-s001.zip › molecules-2902114-supplementary.pdf]

Investigations of major α-dicarbonyl content in U.S. honey of different geographical origins

Kate Nyarko[1] and C. Michael Greenlief [1,*]

[1] University of Missouri-Columbia, Department of Chemistry, Columbia, MO, 65211, USA

[*] Corresponding author: greenliefm@missouri.edu: Tel: +01-573-882-3288

Table S1: Retention time, chemical formulas, measured mass, and fragment masses of MGO, GO and 3-DG derivatives.

α-dicarbonyl derivative	Chemical formula	Measured mass, Da	Retention time, min	Product ions, Da
MGO	$C_9H_8N_2$	145.2	7.65	118.1, 126.8, 134.8
GO	$C_8H_6N_2$	131.2	6.50	98.1, 102.1, 84.1
3-DG	$C_{12}H_{12}O_3N_2$	235.3	5.45	217.1, 199.1

Table S2: Information on honey samples collection

Honey supplier	Country of origin	Floral source	Year
New Zealand Co Ltd Wanaka	New Zealand	*Leptospermum scoparium*	2023
Local hive company	USA (Washington, Texas, Southern California)	N/A	2023

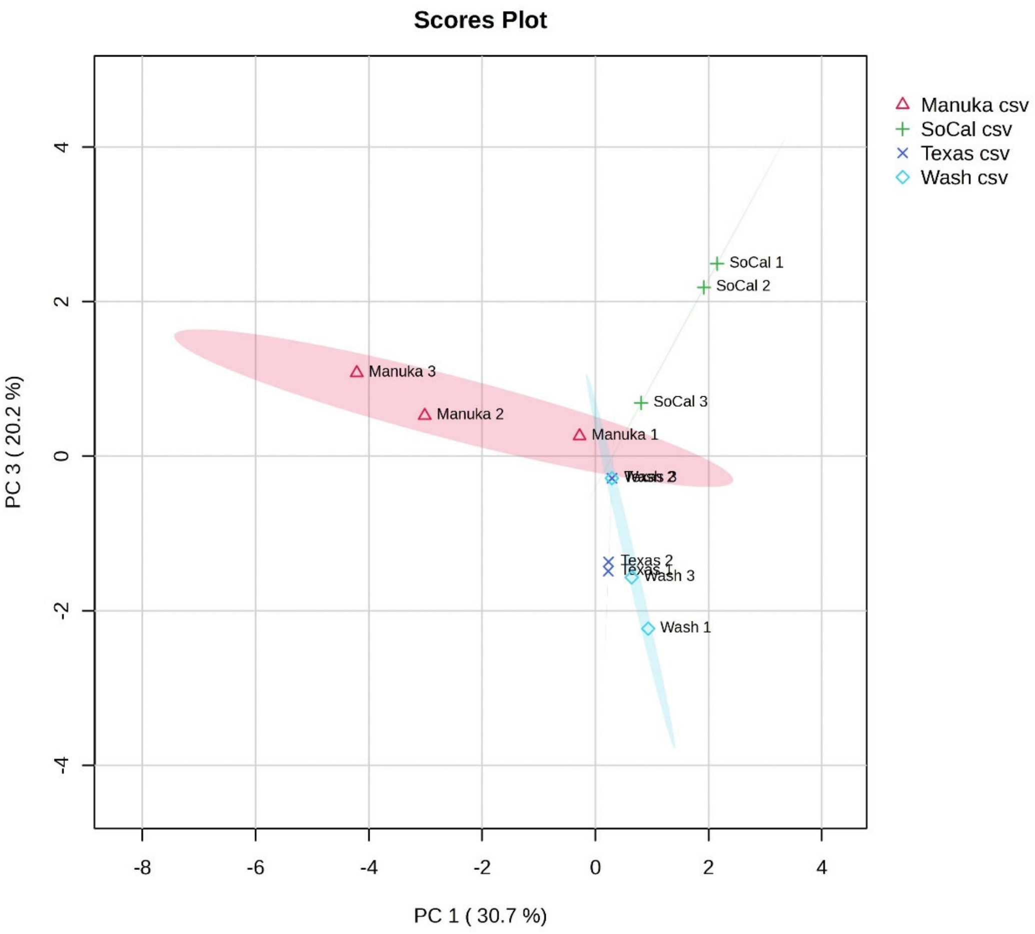

Figure S1: PCA plot showing the geographical origin variations for U.S. honey samples and Manuka honey from New Zealand.
